# How is COVID-19 Affecting the Mental Health of Children with Special Educational Needs and Disabilities and Their Families?

**DOI:** 10.1007/s10803-020-04577-2

**Published:** 2020-07-31

**Authors:** Kathryn Asbury, Laura Fox, Emre Deniz, Aimee Code, Umar Toseeb

**Affiliations:** Department of Education, University of York, York, YO10 5DD UK

**Keywords:** COVID-19, Special educational needs, Disabilities, Mental health, Parents

## Abstract

Parents of children with Special Educational Needs and Disabilities in the UK (*n* = 241) were asked to describe the impact of COVID-19 on their own mental health and that of their child. An inductive content analysis of the data was undertaken. Both parents and children appear to be experiencing loss, worry and changes in mood and behaviour as a result of the rapid social changes that have occurred. Some parents reported feeling overwhelmed and described the impact of child understanding and awareness. Finally, a minority of parents reported that COVID-19 has had little impact on mental health in their family, or has even led to improvements. Implications for how to support these families in the immediate future are discussed.

## Introduction

The need to protect vulnerable people from COVID-19, and not overwhelm the health service, has led to major social change in the UK over a very short period of time. Since 23rd March 2020, UK residents have been required to stay at home and observe social distancing. In cases where they have symptoms of COVID-19, or are particularly vulnerable to its effects, they are required to fully self-isolate. At the same time, UK schools were closed to all but keyworkers’ children and vulnerable groups. These vulnerable groups include some children with Special Educational Needs and Disabilities (SENDs), particularly those with an Education and Health Care Plan (EHCP). However, due to the risks involved, many families with a child eligible to attend school have not chosen to take up a place.

There are good reasons to believe that this pandemic, and our response to it, may affect children with SENDs and their families disproportionately, and that this may have negative implications for their mental health. Some mental health problems are known to be associated with SENDs in normal circumstances, such as anxiety in Autism Spectrum Conditions (ASCs: van Steensel and Heeman 2017). It seems likely that the scale and speed of the social change that has occurred since 23rd March could exacerbate existing mental health problems and trigger new ones.

Families who are raising children with SENDs face more stressors, on average, than those with neurotypical children (McConnell and Savage 2015; McStay et al. 2014). Such stressors can influence the quality of family relationships, making patient and empathic parenting challenging at times (Osborne et al. 2008). Teachers also experience stress in working with children with SENDs, leading to a pattern of some burning out and leaving special education (Brunsting et al. 2014), with some research suggesting that perceived support may be a protective factor (Langher et al. 2017).

Staying at home, and in most cases not attending school, creates a uniquely stressful situation for children with SENDs and their families. Carefully developed routines have been disrupted; support networks have disintegrated; and parents have been asked to do a job that trained teachers find challenging, without any training. These changes have happened abruptly and the consequences could be particularly profound in the SEND community. It is therefore important to ask how COVID-19 is affecting the mental health of these families, with a view to gaining insight into how schools and society can support them over the coming months.

## Methods

### Ethics

Ethical approval for this study was granted by the Department of Education Ethics Committee, University of York (Reference: 20/05).

### Participants

Participants were 241 parents or carers of school-aged children with SENDs in the United Kingdom (*M* age = 9 years; range 5–18). Of the children, 71% were boys; 88% were White British (remainder: 6% Mixed, 3% Asian, 2% White non-British, 1% Other); 44% were in mainstream schools and 70% had an EHCP. School places were made available to 73% of the sample but only 8% had taken up their place. The range of SENDs that were represented can be seen in Table 1.

**Table 1:** Type of special educational needs and disabilities as reported by the parent

Type of SEND	N (%)
Autism Spectrum Conditions	197 (82%)
Attention Deficit Hyperactivity Disorder	56 (23%)
Attention Deficit Disorder	15 (6%)
Developmental Coordination Disorder	24 (10%)
Developmental Language Disorder	45 (19%)
Dyslexia	21 (9%)
Global Developmental Delay	12 (5%)
Physical Disability	14 (6%)
Speech Disorder or Impediment	29 (12%)
Social, Emotional, and Mental Health Difficulties	76 (32%)
Sensory Processing Disorder	11 (5%)
Visual Impairments	12 (5%)
Other^a^	44 (18%)

Parents were asked to select all that applied to their child from a list

^a^Table only includes types of special educational needs and disabilities that were endorsed by > 10 parents. The remainder were included in the other category, which includes conduct disorder, dyscalculia, dyspraxia, Down syndrome, epilepsy, hearing impairment, and moderate learning difficulties

Of the 241 parents/carers who provided data on their own and their child’s mental health, 92% were mothers, 95% were from England (remainder from Scotland and Wales) and 63% had a pre-tax household income of less than £40,000 (approximate UK median income). Participants were recruited between 22nd March and 1st April 2020 during the first fortnight after school closures, via existing research networks, special schools and online platforms.

### Measures

As part of a wider project on the impact of COVID-19 on families with children with SEND, participants were asked to answer the following free response question: Please describe in your own words how the coronavirus outbreak is affecting your mental health and your child’s mental health.

### Coding and Analysis

An exploratory, inductive content analysis was undertaken (Bengtsson 2016). As an initial step all five authors independently coded data from the same 15 participants and discussed this ‘open coding’ process in detail. On the basis of these discussions, the authors independently coded data from a further 15 participants and, following detailed discussions, agreed codes. This process was used to develop a codebook made up of 48 codes (see Table 2). Some of these codes were specific to parents e.g. Anxiety (parent) and Anxiety (child), recognising that at some points participants were describing their own experiences and at others reporting on their child. We divided the remaining data (from 211 participants) between three researchers who each used the codebook to code one-third of the data. A second rater independently coded 20% of the data, selecting every fifth participant in all three batches of data (*n* = 43). We tested for intercoder reliability using Fleiss’ kappa and the results of this analysis are shown in Table 2.

**Table 2:** Intercoder reliability for first cycle coding

CODE	Fleiss’ Kappa	Frequency	Confidence intervals
Anxiety (P)	0.95	27	0.94–0.96
Distress (P)	1.00	6	0.99–1.01
Fear (P)	0.69	11	0.68–0.70
Low mood (P)	0.72	8	0.72–0.73
Stress (P)	0.77	10	0.76–0.78
Boredom (P)	1.00	2	0.99–1.01
Overwhelmed (P)	0.85	16	0.84–0.86
Anger (P)	*	0	*
Positive emotions(P)	0.79	5	0.78–0.80
No/low impact (P)	1.00	2	0.99–1.01
Lonely (P)	*	0	*
Frustration (P)	*	0	*
Uncertainty (P)	0.48	4	0.47–0.49
Always together (P)	0.72	8	0.72–0.73
Food (P)	1.00	2	0.99–1.01
Concern for child’s future (P)	0.77	10	0.76–0.78
Loss of specialist support (P)	1.00	4	0.99–1.01
Total responsibility (P)	0.48	4	0.47–0.49
Communication (P)	*	0	*
Self-efficacy (P)	0.64	6	0.63–0.65
Competing responsibilities (P)	0.84	7	0.84–0.85
Changes in routine (P)	1.00	2	0.99–1.01
Finance (P)	− 0.01	1	− 0.02 to 0.00
Loss of community (P)	*	0	*
School input (P)	0.66	3	0.65–0.66
Under pressure (P)	1.00	2	0.99–1.01
Anxiety (C)	0.90	34	0.89–0.91
Fear (C)	0.72	8	0.72–0.73
No/low impact (C)	0.64	6	0.63–0.65
Stress (C)	0.84	7	0.84–0.85
Low mood (C)	1.00	4	0.99–1.01
Frustration (C)	0.79	5	0.65–0.66
Distress (C)	1.00	10	0.99–1.01
Confusion (C)	1.00	4	0.99–1.01
Challenging behaviour (C)	0.76	22	0.75–0.76
Positive emotions (C)	1.00	6	0.99–1.01
Boredom (C)	1.00	4	0.99–1.01
Behaviour change (C)	0.63	9	0.62–0.64
Sleep (C)	− 0.04	3	− 0.05 to − 0.03
Change in routine (C)	0.72	25	0.71–0.73
Awareness (C)	1.00	6	0.99–1.01
Understanding (C)	0.92	15	0.91–0.93
Friends (C)	1.00	6	0.99–1.01
Food (C)	*	0	*
Communication (C)	− 0.02	2	− 0.03 to − 0.01
Motivation (C)	− 0.01	1	− 0.02 to 0.00
Loss of community (C)	0.36	5	0.35–0.37
School input (C)	0.66	3	0.65–0.66

*Kappa was not calculated because the code was not used in the reliability checking sample on n = 43. Neither rater applying it to any participant implies perfect agreement

We found moderate to strong intercoder agreement for most of the 48 codes applied to the data. Fleiss’ kappa measures the level of agreement over and above chance with 0 suggesting that agreement is no better than chance and 1 indicating perfect agreement. Altman (1999) suggests that κ < 0.20 is poor, 0.21–0.40 is fair, 0.41–0.60 is moderate, 0.61–0.80 is good and 0.81–1.00 is very good. Table 2 shows that agreement on 41 of the 48 codes was either very good (26) or good (15). Of the remaining seven codes agreement on three was moderate/fair and agreement was poor for four. This was largely explained by these codes being applied in few cases (< = 3), exaggerating the effects of disagreement. The second rater reviewed the full dataset to ensure that the seven codes with agreement that was neither good nor very good were applied consistently and discussed areas of disagreement with the original coders, achieving group consensus through this team discussion process (Harry et al. 2005). Final tweaks were made to the codebook and the agreed codes were clustered into categories. Our analysis was manifest rather than latent, attempting to stay close to the participants’ own words rather than identifying underlying meanings (Braun and Clarke 2013).

## Results and Discussion

The frequencies for the agreed codes are shown in Table 3. It is clear that a large proportion of families report that COVID-19 has affected their mental health, often leading to an increase in anxiety and fear. Smaller numbers also reported increases in distress, low mood and stress. The data suggests that more parents than children have experienced increased anxiety (44% vs 25%) and stress (12% vs 5%), although it is important to note that this is parent-reported data, but frequencies for parent-reported fear, distress and low mood were similar for parents and children.

**Table 3:** Frequencies for final agreed codes

Code	Sub-code	Frequency
Anxiety (P)		106
	Anxiety for self (P) Anxiety for others (P) Concern for child’s future General anxiety (P)	*4* *17* *27* *58*
Change in routine (C)		84
Anxiety (C)		60
	Anxiety for self (C) Anxiety for others (C) General anxiety (C)	*7* *5* *48*
Challenging behaviour (C)		44
Understanding (C)		40
	Limited understanding helps (C) Limited understanding hurts (C) Good understanding helps (C) Good understanding hurts (C)	*2* *34* *2* *2*
Fear (P)		34
	Fear for self (P) Fear for others (P) General fear (P)	*6* *9* *19*
No/low impact (C)		33
Fear (C)		31
	Fear for self (C) Fear for others (C) General fear (C)	*7* *7* *17*
Overwhelmed (P)		30
Loss of friends and community (C)		29
	Friends (C) Loss of community (C)	*14* *15*
Stress (P)		28
Specialist input (P/C)		26
	School input (P/C) Loss of specialist support (P/C)	*14* *12*
Positive emotions (C)		24
No/low impact (P)		23
Distress (C)		22
Awareness (C)		19
	Limited awareness helps (C) Limited awareness hurts (C) Good awareness helps (C)	*7* *9* *3*
Competing responsibilities (P)		19
Changes in routine (P)		19
Behaviour change (C)		18
Low mood (P)		18
Uncertainty (P)		17
Self-efficacy (P)		16
Distress (P)		15
Low mood (C)		14
Boredom (C)		13
Positive emotions (P)		13
Always together (P)		12
Finance (P)		12
Confusion (C)		11
Communication (P)		11
	Good communication (P) Poor communication (P)	*3* *8*
Motivation (C)		11
Stress (C)		11
Total responsibility (P)		10
Frustration (C)		10
Sleep (C)		10

In all cases (P) denotes that the code relates to the parent/carer and (C) denotes that the code relates to the child. We only report codes that were mentioned at least 10 times (other than sub-codes). The 9 codes that were excluded on this basis (with frequencies in brackets) are: Boredom (P,7); Lonely (P,5); Frustration (P,5); Food (P,5); Food (C,5); Communication (C,3); Loss of Community (P,6); Anger (P,2); Under Pressure (P,4)

Our final 35 codes were organised into six categories that represent the impact of COVID-19 on mental health as reported by these SEND families: Worry; Loss; Mood, Emotions & Behaviour; Knowing what is going on; Overwhelmed and Minimal or Positive Impact. Table 4 shows that codes were organised into categories—groups of codes that clustered together. For example, the category of worry is made up of three sub-categories (worry for self, worry for others and general worry) which, in turn are made of clusters of codes e.g. worry for self represents four codes, namely, parent and child anxiety for self and parent and child fear for self. Clustering codes into categories is the process via which the researchers identified patterns in the data (Bengtsson 2016).

**Table 4:** Categories, sub-categories, codes and examples

Category	Sub-category	Codes	Meaning unit
Worry	Worry for self	Anxiety for self (P and C) Fear for self (P and C)	I have 2 children with ASD and one has OCD—my eldest son. He’s beside himself constantly washing. This child was off school the week before we were told to as he was sure he was going to die. Was using a hand sanitiser a day. Irrational thinking. Has become even more aggressive and curls up repeating I’m scared I’m just scared He will not leave his room, he’s worried the family has it and will give it to him
	Worry for others	Anxiety for others (P and C) Concern for child’s future (P) Fear for others (P and C)	My son is very worried about seeing his grandparents as he feels he may infect my father who has underlying health issues. He tells him he will go and see him and then doesn’t want to go I worry I’m not doing home Ed correctly, and that my son will fall even further behind at school due to my failings He only eats a limited amount of very specific things which means we have to shop almost daily, putting ourselves at risk more than we would if we didn’t have to. Unlike other children, my boy will NOT eat if he’s hungry enough. He will starve! I was fine until the government implemented the who lives who dies policy this has made me very worried and anxious about if my children do require care if they will get it
	General worry	General anxiety (P and C) General fear (P and C) Sleep (C)	… she’s wanted to sleep comfortably curled up in a large plastic storage box (on the floor next to her bed) for a few nights now instead of her bed. She wants to spend a large part of her days watching her tablet inside of the box too My son’s anxiety is sky high, his hands and arms are red raw from constant washing despite me frequently applying e45. He has started to pick at the dry skin making it worse
Loss	Loss of support networks and structures	Loss of friends and community (C) Boredom (C)	Our entire support network has been taken away from us, including family. I am a single parent of twins, both who have complex learning difficulties and the thought of having to manage alone fills me with dread …his world has been turned upside down, no school, no church, no music lessons, no karate, no seeing friends and family, and he doesn’t understand why She's also bored at home as she thrives from contact with staff and pupils
	Loss of specialist input	School input (P/C) Loss of specialist support (P/C)	We have received no support from my SEN school and I don’t know his routine or how to meet his needs … Homeschooling is difficult as the work sent from school isn’t appropriate (his sister is 4 years younger and they sent home exactly the same work, which is no good for his self-esteem) … The pandemic makes me worry for my son as I fear he will fall behind even further without the specialist teaching or having access to his speech and language therapist It looks like special needs students are left with no support in place and they have been discriminated by not providing them online classes or worksheets like their neurotypical peers and this has left so many parents in limbo
	Loss of routine	Change in routine (P and C)	Lack of structure and the routine of school has meant that B is more anxious, hyper and lashing out. B will not attempt school work at home as home is home and school is school and they do not mix We have had to reorganise businesses, mortgages, 20 staff wages, taking no income for ourselves next month so preparing for that, cancelling financial arrangements, taking food and medication to elderly parent, sorting out child with Down syndrome who’s residential school have a pupil tested positive for CV, whilst cooking, baking with children, reading and cleaning constantly …
	Financial loss	Finance (P)	I had just set things up so that I could have some respite and time to work from home but now my job is gone (zero hours contract) …
Mood, emotions and behaviour	Feeling down	Low mood (P and C) Distress (P and C)	My son is crying all the time and said that he cannot handle all this. He has started talking to people who are not there Last night he said he wants to die but couldn’t say why Like I’m drowning and nobody can see me
	Acting out	Challenging behaviour (C) Frustration (C)	My son will not stay in everyday—it’s so difficult. My daughter has reported him to the police My son is being more disruptive at home. Wiping food into the carpet and over the walls, breaking toys and ripping up books He misses school terribly and this shows in his behaviour which I am struggling with. He throws any object he can get his hands on and throw across the room. He bites himself and me, screams and cries running around the house
	Behaviour change	Behaviour change (C) Motivation (C)	My daughter has started stimming constantly, has constant violent outbursts (which she doesn’t have often usually) and has started having night terrors … upset that all the work gone into GCSE has been wasted, finding it hard to get motivated doing any work when GCSEs will now be decided by a panel …
Knowing what is going on	Positive implications	Self-efficacy (P) Limited awareness helps (C) Limited understanding helps (C) Good awareness helps (C) Good understanding helps (C) Good communication (P)	My son asked some initial questions which I answered about the outbreak, his school language unit were also good in supporting the children to understand, and he is not currently worried about it We are very fortunate that R is very clever and if you explain something in enough detail that can really allay a lot of her stresses
	Negative implications	Confusion (C) Uncertainty (P) Limited understanding hurts (C) Good awareness hurts (C) Good understanding hurts (C) Poor communication (P)	My daughter doesn’t understand why she can’t see her family and that’s made her feel quite sad and lonely, sometimes thinking it’s a punishment for something she might have done … My son has no idea why his whole world has been turned upside down and why none of the things he loves are … available to him
Overwhelmed	Too much	Total responsibility (P) Competing responsibilities (P) Overwhelmed (P) Always together (P)	Working from home and having a child with SEN is near enough impossible. Affecting our mental health negatively I have felt completely unprepared for the reality of dealing with her 24/7 without any support or respite, as her behaviour is extremely challenging and she can become very physical Two kids, one me. It’s tough. My mental reserves are draining fast
	Stressed	Stress (P and C)	I can honestly say I have never been stressed or felt overwhelmed before, because for the first time in my life I now do. I have found I am having trouble breathing and filling up my lungs, no matter how many deep breaths I take
Minimal or positive impact	Positive emotions	Positive emotions (P and C)	… in a lot of ways things have been easier, I don’t feel like I have to push my child into doing activities and day trips etc. just to keep up with everyone else. I feel less under pressure to battle with her to get her to go to the supermarket or cinema or park etc. as she always finds these a struggle noisewise and she doesn’t like being near other people so being isolated has taken away a lot of the stress … his anxiety is much less than normal as he no longer has the daily torture of going to school
	Minimal impact	No/low impact (P and C)	The isolation part is nothing new for us because my son doesn’t have many friends and we don’t get out much anyway

N.B. Participants are quoted verbatim except where it was clear that a typographical error had been made or where a minor grammatical tweak would aid understanding without altering the meaning

*Worry* was sub-divided into three sub-categories: worry for self, worry for others and general worry. Although some of the worry described is likely to be shared by parents and children in general, the majority was specific to families with a child with SENDs. The examples given in Table 4 illustrate the point that participants often described extreme anxiety reactions that are not likely to be commonplace in the general population, and that are characterised by known features of SENDs e.g. restricted food preferences. Also, parental worry for others, particularly concern for their child’s future, focused on their children falling even further behind in school because they did not know how to meet their needs, and worries about who would look after them if they (the parent) died as a result of COVID-19. The level of worry many SEND families report appears to be substantial and serious, and supporting these families in ways that will help to alleviate or reduce their anxiety should be a priority for education, health and social care professionals.

*Loss* was also described by many participants as a result of COVID-19, and these losses were organised into four sub-categories: loss of routine, loss of support network and structures, loss of specialist input and, for a minority, financial loss. Some of these losses are likely to be widespread in the community, such as loss of access to support networks, changes in routine and financial losses. However, the data suggests that, in some cases, the effects of these widespread losses are amplified in families with a child with SENDs because the challenge of meeting the child’s needs is simply greater. Also, for some children with SENDs it is not possible to explain why these losses have occurred, creating further difficulties. We have several examples in the data of lone parents who are isolated with a child who displays very challenging behaviour without access to any of the support and respite that usually helps them to fulfil their parental role effectively. These lone parent SEND families appear particularly vulnerable in the current situation. Furthermore, there is a suggestion that some parents feel that during the first fortnight of school closures their children were insufficiently supported. In some cases this was because no tailored support was offered (e.g. children in mainstream classrooms being provided with the same input as the rest of the class). Parents also mentioned a pressing need for some children to see familiar faces, such as those of their teacher or teaching assistant, a need that while shared by some neurotypical children is likely to be amplified for children with SENDs such as ASCs.

*Moods, emotions and behaviour*, including low mood, acting out and behaviour change, were mentioned by many participants. While low mood and distress are likely to be widespread the data suggests they may be experienced more severely within the SEND community. The types of challenging behaviour described in Table 4 are clearly difficult for both parents and children to cope with, involving police, violence to self and others and a level of destructiveness that is likely to be uncommon among children of equivalent ages without SENDs. The distress this causes suggests that support is needed before crisis point is reached in some families.

*Knowing what is going on* was a key element of some participants’ responses. Parents described situations in which a child’s low level of understanding led to distress because they could not understand why everything had changed. In the cases of minimally verbal children their disorientation was sometimes expressed in challenging behaviour. The data suggests that better understanding was associated with better outcomes. One way to address this issue may be to prioritise children known to have limited understanding and to create resources that may help to enhance their understanding, such as social stories, while working to ensure that those aspects of life that *can* stay the same (or similar) *do* stay the same (or similar) e.g. providing remote face to face support. Child awareness of the situation was described as having both positive and negative effects in that while some children seem oblivious to COVID-19 and have not minded the changes, others are frustrated for the reasons described above.

*Overwhelmed* was the penultimate category used to describe participants’ responses. A substantial minority of parents described themselves as being stressed by the new demands that were placed on them, including meeting all of their child’s special or additional needs without support, a break or respite, often alongside working and meeting the needs of others in the family. Given that this data was collected within two weeks of school closures we might expect this situation to worsen over time although it also remains possible that, as families become more acclimatised to the situation, they may become less overwhelmed or there may be no discernible change.

*Minimal or positive impact* was described by a substantial minority of families for whom the impact of COVID-19 was not perceived as harmful. It was notable that positive emotions were often expressed in families where the child has a hard time at school and feels safest at home. For these children, self-isolation and social distancing may lead to a period of calm respite, creating a more relaxed environment for them and their families.

## Conclusion

There is evidence in the literature that children with SENDs and their families are likely to be at greater risk of experiencing poor mental health, and to be under substantially greater pressure, than less vulnerable families during COVID-19. The data presented here provides some support for this likelihood, although not definitive support as the study did not include a comparison group of neurotypical children and their families. It is therefore important to assess the specific needs of these families and take concrete steps to meet them. These families were also asked what support they would like during COVID-19 and their responses are reported elsewhere (Toseeb et al. 2020). Frequently mentioned suggestions included specialist professional advice for parents focused on how to meet their child’s educational and mental health needs; setting appropriate tasks and resources for home learning; and providing opportunities to see familiar faces, albeit remotely. The data presented here, from the same group of families, indicates that support along these lines would be beneficial to many families with a child who has SENDs. Also, it is important to note that some families will not need or want additional support but that identifying those who are struggling, and providing tailored support, should represent a priority for education, health and social care during the coming weeks.

## Data Availability

Deidentified individual participant data will not be made available at this stage due to ongoing data collection but will be made available once data collection is complete.
